# A Targeted Metabolomics MRM-MS Study on Identifying Potential Hypertension Biomarkers in Human Plasma and Evaluating Acupuncture Effects

**DOI:** 10.1038/srep25871

**Published:** 2016-05-16

**Authors:** Mingxiao Yang, Zheng Yu, Shufang Deng, Xiaomin Chen, Liang Chen, Zhenyu Guo, Hui Zheng, Lin Chen, Dingjun Cai, Bo Wen, Qiaofeng Wu, Fanrong Liang

**Affiliations:** 1 College of Acupuncture and Tuina, Chengdu University of TCM, Chengdu, 610075, China; 2 Metabolomics, Scientific Technology Department, BGI, Shenzhen, 518083, China

## Abstract

The critical role of metabolic abnormality in hypertension is increasingly recognized, but its biomarkers are not clearly identified. In this study, 47 chemical compounds recorded by literature were employed as target metabolites of essential hypertension (EH). We detected their content in the plasma of EH patients and healthy subjects by using the Multiple Reaction Monitoring-Mass Spectrometry (MRM-MS). After screening the most altered compounds, acupuncture was used to treat patients for 3 months and these plasma metabolites were tested again. The results showed that oleic acid (OA) and myoinositol (MI) were the most important differential metabolites between the hypertensive plasma and the healthy plasma. They were also closely correlated with 24-hour blood pressure and nocturnal dipping. Moreover, plasma OA and MI could be restored to normal levels by acupuncture, accompanying with reduction of 24-hour systolic and diastolic blood pressure [from 145.10 ± 9.28 mm Hg to 140.70 ± 9.59 mm Hg (P < 0.0001), and 88.35 ± 7.92 mm Hg to 85.86 ± 7.95 mm Hg (P = 0.0024), respectively] and improvement of circadian blood pressure rhythm. This study demonstrated that plasma OA and MI were potential hypertension biomarkers and they could be used to preliminarily assess the treating effects such as acupuncture.

Hypertension is a leading risk factor for cardiovascular, cerebral, and renal events. It accounts for at least 45% of deaths due to heart disease and 51% of deaths due to stroke^1^. However, despite its high prevalence, only 53.7% of hypertensive adults are satisfactorily treated^2^, and the successful control rate of hypertension is only 8.1%^3^. Recently, a large number of studies have linked hypertension to metabolism dysfunction or the metabolic syndrome. Obvious pathology was identified in serum metabolic profiles after the development of hypertension^4^; two-thirds of essential hypertension patients were found to have abnormal glucose metabolism^5^. The metabolic syndrome as a matrix of metabolic dysfunctions amplifies cardiovascular risk associated with high BP^6^. Thus, the role of metabolic abnormalities in the pathology of essential hypertension is increasingly essential. To explore its metabolic profile (targeted or untargeted) perturbations under different environmental or physicochemical conditions may provide us with new perspectives on this disease and may, hopefully, inform more targeted treatment in the future. On the other hand, although many studies have concluded that hypertension is a disease or syndrome involving metabolic disorder, few studies have evaluated which metabolites or chemicals are potential biomarkers for this disease, and fewer still have made use of the disorder’s metabolites to evaluate the effectiveness of different kinds of treatments.

Therefore, in this study, we employed Multiple Reaction Monitoring-Mass Spectrometry (MRM-MS), a new high-throughput method, to detect 47 kinds of low molecular weight plasma metabolites that have already been reported or considered as potential target molecules in the study of hypertension. We chose acupuncture, one of the signature treatment methods of traditional Chinese medicine^7^, to manage hypertension and re-detected these target molecules. By this way, we hope to preliminarily assess the essential hypertension biomarker(s) and their relation to the treating effects such as acupuncture.

## Results

### Baseline information

A total of 113 eligible patients with mean age of 59.82 ± 8. 95 (maximum age 69 years, minimum age 42 years) and 15 healthy subjects with mean age of 55.93 ± 6.30 (maximum age 66 years, minimum age 42 years) were enrolled in the current study. The baseline characteristics and BP-related parameters such as gender, age, dietary habit, nationality, and other physiological parameters like blood sugar, total cholesterol, ALT, AST, BUN, sCr, HDL-C, and LDL-C demonstrated no significant difference in the two groups. However, BP was significantly different between EH patients and the healthy control, as shown in Table 1.

### Oleic acid and myo-inositol are hypertension symptom-related metabolic biomarkers

**Figure 1:**
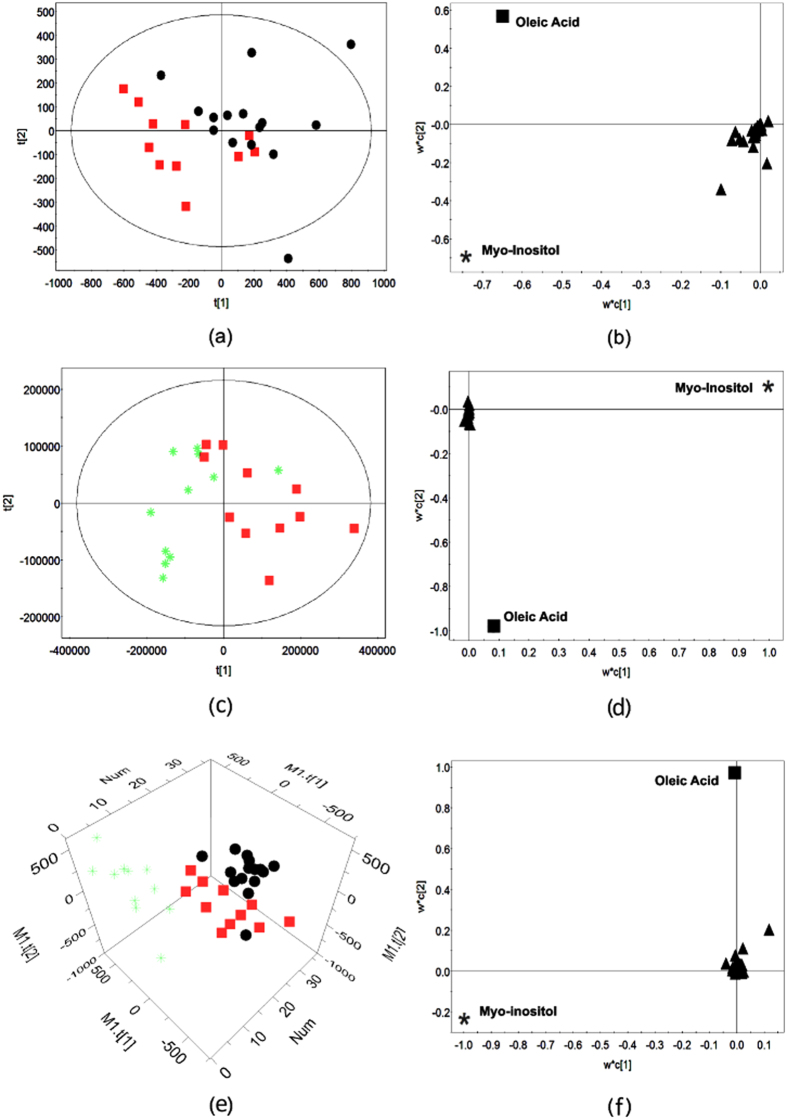
OA and MI as bio-markers of hypertension. For the scatter plot (**a,c,e**), the symbol 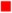 represents baseline EH plasma, the 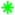 represents post-treatment EH plasma, and the 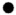 represents healthy control. For loading plot (**b,d,f**), the symbol 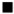 represents oleic acid, the 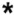 represents myo-inositol, and the 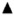 represents other indistinguishable metabolite. (**a**) Hypertension patients and health controls were clearly separated in score plot; (**b**) OA and MI were the most important metabolites distinguishing hypertension plasma from health plasma in loading plot; (**c**) post-treatment hypertension plasma with reversed metabolic abnormalities was separated from pretreatment plasma in score plot; (**d**) OA and MI were the most important metabolites distinguishing pre- and post- treatment plasma in loading plot; (**e**) post-treatment, baseline plasmas of hypertension patients and plasma of healthy controls were clearly separated by 3-D score plot; (**f**) OA and MI were the most important metabolites distinguishing three groups’ plasma in loading plot.

**Figure 2:**
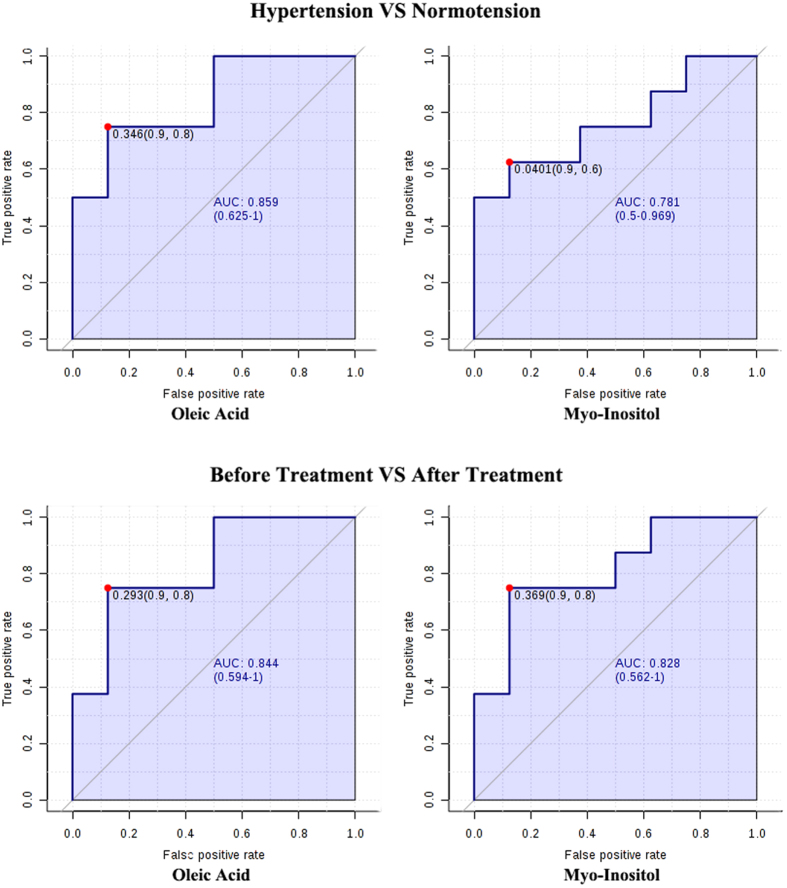
Receiver operating curve analysis for OA and MI. In the comparison between hypertension group and control group, the area under the curve (AUC) of OA and MI is 0.859 (0.625–1) and 0.781 (0.5–0.969), respectively. The AUC of OA and MI is 0.844 (0.594–1) and 0.828 (0.562–1) when comparing the pretreatment data with post-treatment data.

Univariate analysis revealed that among the 47 metabolites, the concentrations of citrulline, D-(+)-galactose, Glycine, fructose, L-tyrosine, OA, MI, and urea were significantly changed in EH patients (compared with control group, P < 0.05, Fold-Change-value > 1.2 or <0.8). Multivariate analysis by OPLS-DA analysis revealed that EH patients and healthy control subjects could be separated by the target metabolites (Fig. 1a). The corresponding loading plots showed that OA and MI were obviously deviated to the origin, indicating OA and MI were two key metabolites that mostly contributing to the separation of EH patients and the healthy subjects (Fig. 1b). The VIP score is 3.91 and 3.70 for OA and MI respectively. The receiver operating curve (ROC) analysis for hypertension patients *vs.* healthy controls showed that the area under the curve (AUC) of OA is 0.859 (0.625–1), and 0.781 for MI(0.5–0.969) (Fig. 2).

Pearson correlation analysis showed that OA was positively correlated with 24-hour systolic BP (R^2^ = 0.25), and MI was inversely correlated with 24-hour systolic (R^2^ = 0.22) and diastolic BP (R^2^ = 0.16). These two metabolites also showed correlation with systolic and diastolic BP nocturnal dipping. The OA showed a negative correlation with SBP nocturnal dipping (R = −0.44) and DBP (R = −0.37), while MI was positively correlated with SBP nocturnal dipping (R = 0.35) (Table 2). Besides, there was a significant positive correlation between 24-hour systolic BP and sucrose, or cellobiose, which suggests that the escalation of 24-hour systolic BP and increases in sucrose, or cellobiose concentration might be affected each other. For 24-hour diastolic BP, there was a significant negative correlation with the concentration of urea and a significant positive correlation with oxaloacetic acid and galactose.

### Plasma oleic acid and myo-inositol of hypertension patients could be successfully restored to normal levels by acupuncture

**Figure 3:**
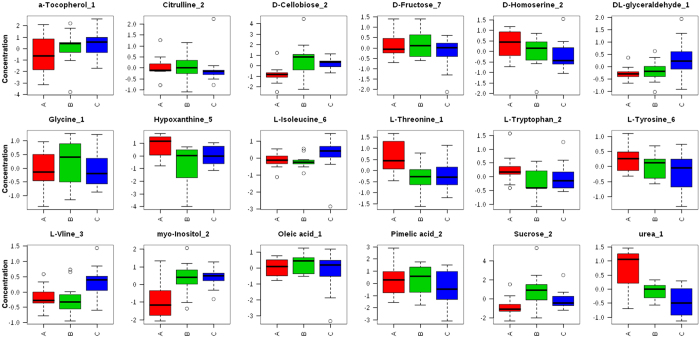
Normalized concentration for key metabolites in hypertension patients’ plasma and healthy subjects’ plasma. Metabolite concentration for several targeted key metabolites were median-normalized and Log10-transormed. (**A**) Is abbreviated for ‘after treatment’, (**B**) is short for ‘before treatment’, and (**C**) for ‘control’.

After finding OA and MI are the two potential biomarkers in EH patients. We used them to evaluate the effect of acupuncture treatment. Interestingly, the OPLS-DA results demonstrated that acupuncture could successfully restore the level of OA and MI to normal levels (Fig. 1c,d). VIP for MI is 5.36 and for OA is 0.55. We performed OPLS-DA analysis for pretreatment plasma, post-treatment plasma, and healthy plasma. The results indicated that these groups could be well separated. (Fig. 1e,f). A VIP analysis using MetaboAnalyst assured that MI (VIP score: 4.24) and OA (VIP score: 3.32), underwent the most significant change after acupuncture (Supplementary Figure 1a). We also compared their concentrations in the three groups and revealed that both metabolites increased after the onset of hypertension, while acupuncture reduced them to different extents, together with lowering BP (Fig. 3, Supplementary Figures 1b and 2). Moreover, in comparing pretreatment data with post-treatment data, the AUC of OA and MI is 0.844 (0.594–1) and 0.828 (0.562–1), respectively (Fig. 2).

### Acupuncture could regulate Blood pressure and its circadian rhythm, which could also be reflected by the plasma levels of oleic acid and myo-inositol

**Figure 4:**
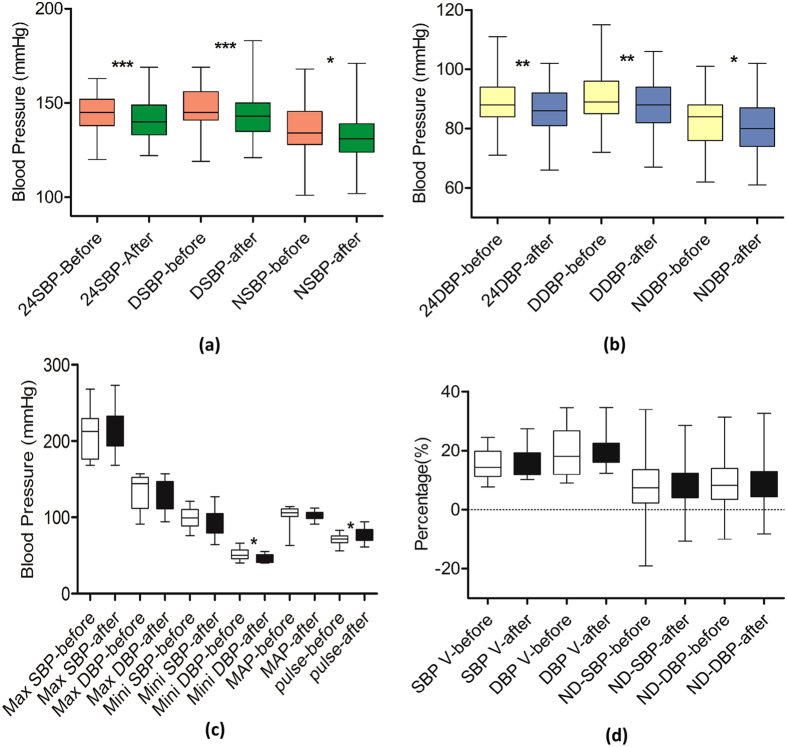
Changes of blood pressure and its circadian rhythm after acupuncture reversing metabolic disorders in OA and MI. (**a**) Systolic BP and (**b**) diastolic BP in baseline and post-treatment; (**c**) max- and mini- BP, mean arterial pressure, pulse and (**d**) BP variability, nocturnal dipping, in baseline and post-treatment.

**Figure 5:**
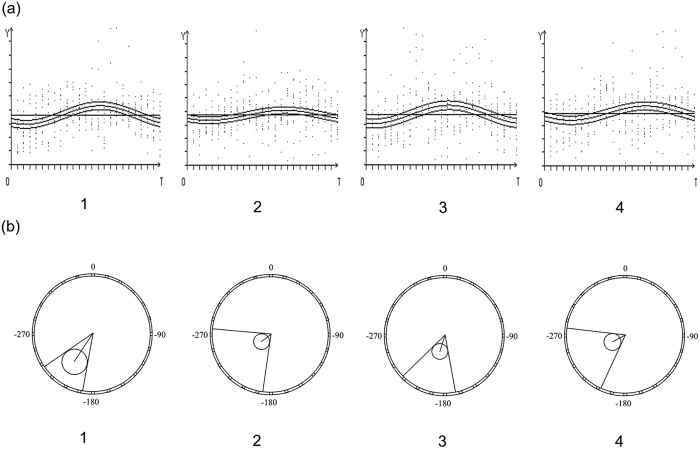
Curves and polar coordinates of circadian variations in BP, 360° = 24 hours, 00:00 = 0°. (**a**) Curve of circadian BP changes for baseline systolic, post-treatment systolic, baseline diastolic, and post-treatment diastolic, respectively; (**b**) Polar coordinate of circadian BP changes in baseline systolic, post-treatment systolic, baseline diastolic, and post-treatment diastolic, respectively.

Acupuncture simultaneously lowered 24-hour BP, improved circadian BP rhythm, along with reversing OA and MI abnormalities. All values of 24-hour, daytime and nighttime (systolic and diastolic) BP, and pulse are regulated by acupuncture (all P < 0.05) (Table 3 and Fig. 4). The 95% CIs on the estimated change from baseline 24-hour BP versus post-treatment were no more than 10 mm Hg (pre- versus post-: SBP: 2.41 to 6.63 mm Hg; DBP: 0.85 to 3.87 mm Hg). The circadian rhythm of BP indicates the increased cardiovascular risk caused by elevated BP. There was no significant change in nocturnal dipping of SBP and DBP after 6-week acupuncture treatment (P > 0.05). However, the Cosinor analysis showed overt BP rhythmicity in both baseline BP and post-treatment BP (P < 0.001). The result also demonstrated that acupuncture changed the mensor, amplitude, and acrophase of BP rhythm. The mensor ± SE (standard error) of BP rhythm was significantly reduced from 145.61 ± 0.95 mm Hg (systolic) and 84.86 ± 0.65 mm Hg (diastolic) to 138.50 ± 1.03 mm Hg and 82.10 ± 0.62 mm Hg. The amplitude of blood rhythm was also reduced. As to the acrophase of BP rhythm, there was a significant reversion from baseline to post-treatment in both SBP and DBP (Fig. 5). After acupuncture treatment, the levels of ALT, BUN were significantly reduced (P < 0.05). As to other parameters, no significant differences were detected in the comparison between pre- and post- acupuncture treatment. No adverse events were reported during the acupuncture treatment course, or after the completion of treatment. The correlation analysis showed that SBP reduction is positively correlated with OA change (R = 0.44) and negatively correlated with MI change (R = −0.20). The nocturnal dipping change in SBP is closely correlated with MI (R = 0.52).

## Discussion

The metabolic abnormality is getting increasingly concerned in the development and treatment of hypertension. A three population-based cohort study demonstrated that identification of metabolic biomarkers of cardiovascular diseases could be helpful for more accurate diagnosis and more precise treatment^8^. In the current study, we found that OA and MI are potential metabolic biomarkers of essential hypertension by using MRM-MS target metabolomics approach. Moreover, the reduction of blood pressure and recovery of its circadian rhythm, induced by acupuncture, were showed to be closely related to these two metabolites.

### Oleic acid and myo-inositol metabolic disorders are closely correlated with hypertension

OA is one of the most important free fatty acids (FFAs), accounting for 40% of FFAs in the healthy blood sample. Elevated plasma levels of FFA are considered to be associated with higher cardiovascular risk, induced by FFA-related oxidative stress in endothelial cells. In addition, OA can help lower the level of low-density lipoprotein (LDL) in the bloodstream, while unchanging the level of high-density lipoprotein (HDL). Although there is no evidence that dietary total OA is closely related to BP in individuals, OA from vegetable sources may contribute to preventing the elevated BP levels in general population^9^. One reason of this might because OA can increase the production of mitochondrial reactive oxygen species and decrease the activity of endothelial nitric oxide synthesis activity^10^. Furthermore, OA can regulate the α and β adrenergic receptors which are involved in controlling the central and peripheral BP^11^,^12^ by modulating monounsaturated fatty acids (MUFA) through the lipid structures of the membrane, cell signaling platforms, and the α_2_-adrenergic receptor pathways. The other key metabolite, MI, is an insulin-sensitizing substance possessing insulin-mimetic properties^13^. In menopausal women with metabolic syndrome, the supplementation of MI helps to manage BP and regulate other metabolic disturbance of cardiovascular risk^14^. The possible mechanism of MI attending in regulating BP may be through inositol 1,4,5-triphosphate receptor (IP_3_R). IP_3_R up-regulation in hypertension is associated with sensitization of Ca^2+^ release and vascular smooth muscle contractility. In hypertension, resistant arteries show elevated responsiveness to vasoconstrictor agonists, and this abnormality relies partly on enhanced Ca^2+^ signaling in vascular smooth muscle (VSM). Studies proved that levels of IP_3_R transcript and protein were significantly increased in mesenteric arteries of hypertensive animals, and pharmacological inhibition of the IP_3_R revealed a higher contribution of IP_3_-dependent Ca^2+^ release to vascular contraction in these arteries. IP_3_R up-regulation in VSM is associated with enhancement and sensitization of IP_3_-dependent Ca^2+^ release, resulting in increased VSM contraction in response to agonist stimulation^15^. Therefore, the results that MI abnormalities in EH patients may indicate IP_3_ changing, which is a crucial mechanism underlying BP elevation.

In addition, we found the levels of plasma OA and MI are independently related to blood pressure because there is no difference seen in other physiological parameters like blood sugar, total cholesterol, ALT, AST, BUN, sCr, HDL-C, and LDL-C between hypertension patients and health control subjects. As we know, high blood pressure is one of the components that results in metabolic syndrome. On the other hand, metabolites such as OA and MI are also related to some metabolic syndrome (such as high cholesterol and diabetes). Thus, whether the alteration of OA and MI is specifically resulted from hypertension is a great challenge. In this study, subjects with abnormal blood sugar, total cholesterol, ALT, AST, BUN, sCr, HDL-C, and LDL-C were excluded according to the inclusion/exclusion criteria. After applying the univariate analysis and the multivariate analysis, OA and MI were two compounds that significantly changed in disease condition as compared with the healthy subject. Moreover, these two contributed the most to separate disease plasma and health ones, as indicated in the PLS-DA analysis. Therefore, OA and MI are proved to be closely correlated with hypertension. However, this does not mean that the alteration of OA and MI in plasma has no relation with diabetes or high cholesterol. One obvious reason is that we have only studied borderline hypertension. Besides, other studies found that OA could induce a synergistic mitogenic response with angiotensin II in vascular smooth muscle cells in obese hypertensive patients, which consequently accelerated vascular disease. The increased OA level and enhanced activity of the renin-angiotensin axis in patients with the risk factor cluster could also interact with atherosclerosis^16^. Similarly, MI is efficient in lowering post-prandial blood glucose^13^ and is involving in insulin resistance and with the long-term microvascular complications of diabetes^13^. Clinic trails have proved it. For instance, obese pregnant women treated by MI, or combined with D-chiro-inositol and folic acids were found to have a significant reduction in systolic BP^17^,^18^,^19^.

### Blood pressure and its circadian rhythm could be mediated by acupuncture treatment, accompanying with modulating the oleic acid and myo-inositol metabolism

Our study demonstrates the improvement in the plasma level of OA and MI can reflect the therapeutic effect of acupuncture to some extent. Acupuncture is a traditional therapeutic treatment for EH patients especially for those on early stage or with complications. Flachskampf’s large clinical trial^7^ showed that active acupuncture significantly reduced both SBP and DBP for at least 5/3 mm Hg. The magnitude of reduction fell between that induced by angiotensin-converting enzyme (ACE) inhibitor and calcium antagonist^20^. Considering that lowering BP by 10/5 mm Hg (systolic and diastolic, respectively) was associated with a reduction in risk of recurrent stroke^21^ and significant reductions in all cardiovascular outcomes, including mortality^22^, the effect of acupuncture could be helpful. A similar treatment effect has been reported with other forms of guideline-recommended antihypertensive interventions^23^. In our study, the magnitude of systolic BP reduction induced by acupuncture therapy was 4.52 mm Hg (95% CI, 2.41 to 6.63), which is in accordance with Flachskampf’s results. Besides, the current study also demonstrated that acupuncture improved the amplitude and acrophase of the circadian rhythm. Interestingly, we found that OA and MI could be used to reflect the anti-hypertensive effect of acupuncture. Firstly, since no plasma biochemical indicators tested (including blood sugar and cholesterol) were changed after acupuncture, the modulating of OA and MI metabolism can reflect the acupuncture’s effect. In addition, the correlation analysis results showing that the SBP reduction was positively and negatively correlated with OA and MI change (respectively) also provide evidence that OA and MI metabolism reflect the antihypertensive effect of acupuncture. Furthermore, although the ROC analysis showed that MI is not as sensitive for hypertension as OA, the curve AUC of OA and MI were 0.844 and 0.828 respectively after acupuncture treatment, validating their sensitivity for evaluating acupuncture effects.

Till now, it is still not clear how those two metabolites are involved in acupuncture’s antihypertensive effect. Recent studies suggested that the hypoglycemic effect of acupuncture is probably attributable to the reduction of FFA^24^,^25^. Another study also demonstrated that acupuncture reduced FFAs in rats with non-alcoholic fatty liver disease, and thereby reduces lipogenesis and liver fat deposition^26^. Therefore, the regulation of FFA metabolism (OA involved) could be an important aspect of acupuncture’s therapeutic effect for hypertension. Regarding the MI and the Inositol phosphate metabolism, several studies shed light on its role in acupuncture’s therapeutic effect. The inositol triphosphate signal transduction pathway is one of the main intracellular second-messenger molecular pathways closely involved in acupuncture’s regulating effects^27^. Under normal conditions, electroacupuncture-induced changes in the IP_3_ level in rat brain and spinal cord, suggesting links to the PI system via its signal transduction pathways. In mild cerebral artery occlusion, acupuncture was shown to downregulate the significant increases in expression of Angiotensin II and influence its receptor-mediated phosphatidyl inositol signal pathway, consequently reducing vasoconstriction and improving blood supply to the ischemic region, and ultimately conferring beneficial effects on cerebral ischemia^28^. In addition, acupuncture has been shown to effectively inhibit cerebral ischemia-induced increased levels of intracellular IP_3_ and diacylglycerol (DAG) in rat cerebral arteries, which may contribute to acupuncture’s effect in modulating vascular constriction and dilation to ameliorate cerebral microcirculation^29^. Thus, the regulation of fatty acid and the inositol pathway could be an important part of acupuncture’s effect on blood pressure.

In conclusion, by a combination of laboratory tests with clinical effectiveness, which is much more comprehensive, OA and MI were found to be closely related to increased BP and possibly circadian BP rhythm disorder in this study. Besides, the restoration of OA and MI in the plasma can reflect the up-regulating BP and the improvement of circadian rhythm which was induced by acupuncture treatment. Future studies are welcome to examine our hypothesis by enlarging the sample size and by involving different drug-related studies.

## Methods

### Ethics, consent and permissions

All procedures were designed according to the Declaration of Helsinki’s^30^ ethical principles for medical research involving human subjects. The study protocol has already been ethically reviewed and approved by Ethics Review Committee of the Teaching Hospital of Chengdu University of TCM ( 2012KL–003). Participants were clearly explained about all procedures and potential risks and benefits from the study before their inclusion. All included participants provided with written inform consent with their personal signatures.

### Participants

All study participants were recruited from the 3^rd^ teaching hospital of Chengdu University of Traditional Chinese Medicine. Essential hypertension (EH) patients and healthy subjects were included if they fulfilled all the following criteria. Inclusion criteria: (1) aged between 40 and 70 years; (2) diagnosed with stage I hypertension at the first visit, not with some type of secondary hypertension such as renal vascular disease, Cushing’s syndrome, hyperadrenocortism, or drug-induced hypertension; (3) free of other complicated cardiovascular, digestive, respiratory, urinary, blood, nervous, endocrine-system, or other severe primary diseases; (4) did not ingest any drugs or herbs during the 15 days before the start of the study; (5) were not participating in any other study; (6) agreed to cooperate with researchers in all research procedures after they were introduced into this study; and (7) provided written informed consent. Exclusion criteria included epilepsy, sleep apnea hypopnea syndrome, depression or anxiety, pregnancy or lactation, or women of reproductive age who intended to conceive within 1 year; any blood laboratory test abnormality; suffering from acute diseases in the past 2 weeks, such as high fever, or gastritis; or being prescribed any drug or being treated with acupuncture within the past three months. Healthy adults of similar age, body mass index (BMI), and gender were recruited as healthy controls. These healthy adults had no organic or functional disorders, were not infected with the common cold or other conditions, and had not taken medications or received acupuncture treatments within the past three months.

### Interventions

Demographic information and regular physiological data were acquired at baseline. The 24-hour ambulatory BP was measured by an oscillometric device (A&D Co., Ltd., Japan TM-2430) within 24 hours before the commencement and the completion of acupuncture treatment. The basic acupuncture compulsory formula included two acupoints: Taichong (LR3) and Renying (ST9). Arbitrary acupoints included Taixi-KI3, Neiguan-PC6, Zusanli-ST36, and Quchi-LI11. No other acupoints or sham acupoints were used in this study. Licensed Chinese acupuncture therapists performing all treatment procedures have at least five years’ experience of acupuncture treatment. All acupoints were punctured with disposable stainless steel needles (0.25 mm × 40 mm; 0.25 mm × 25 mm; Suzhou Hwato Medical Appliance Co., Ltd., Suzhou City, China). Deqi sensation, which is essential to ensure the clinical efficacy of acupuncture, was subsequently induced by manipulations. The hypertensive patients received acupuncture therapy for 30 minutes in each session, three sessions a week, for six weeks. Every two weeks there was a two-day interval without treatment.

### Sample collection

Fasted venous blood (about 5 mL) of the patients was collected into vacutainer tubes with heparin sodium before and after acupuncture treatment at 8:00–9:00 a.m. respectively. Plasma was obtained by centrifugation at 1500 rpm at 4 °C for 15 min.

### Defining targeted metabolites

Metabolites dramatically changed in Hypertension patients or closely related with the pathophysiological mechanisms of the cardiovascular system were identified^31^,^32^,^33^,^34^,^35^,^36^,^37^,^38^. Finally, total of 47 metabolites or compounds were selected for further MRM-MS measurement. The compounds include L-Tyrosine, L-Phenylalanine, L-Threonine, L-(+)-Lactic acid, L-Valine, L-Leucine, L-Proline, Betaine, Palmitic acid, Stearic acid, Glycine, (±)-a-Tocopherol, β-Sitoseterol, L-Tryptophan, DL-glyceraldehyde, Glycocholic acid, oleic acid, eicosanoic acid, hexanoic acid, Heptanoic acid, nonanoic acid, Galactose, Sucrose, Sorbitol, myoinositol, Fructose, Cellobiose, Urea, Isoleucine, Alanine, citric acid, Azelaic acid, Aspartic acid, 4-Hydroxybenzoic acid, Pimelic acid, L-Serine, Hypoxanthine, D-Homoserine, Uric acid, Trimetlylamine oxide, Pentanedioic acid, Allantoin, Linoleic acid, Citrulline, Oxaloacetic acid, and Sorbose andα-ketoglutaric acid. All standard chemicals were LC–MS grade and purchased from Sigma-Aldrich Company (St. Louis, MO).

### MRM-MS analysis

The analyses were performed on a QTRAP5500 mass spectrometer (AB SCIEX, Framingham, MA, USA.) equipped with a Shimadzu UFLC system, which consisted of Shimadzu LC-20AD XR pumps and a SIL-HTC autosampler (Kyoto, Japan). Liquid chromatography-tandem mass spectrometry data were acquired using a 5500 QTRAP triple-quadrupole mass spectrometer (Applied Biosystems/Sciex; Foster City, CA, USA). LC−MS/MS analysis was performed on a 5500 QTRAP triplequadrupolelinear ion trap mass spectrometer (Applied Biosystems/Sciex; Foster City, CA, USA) equipped with electrospray ionization (ESI) source interface operating in positive ion mode. Chromatography separation was achieved on an Eksigentekspert™ microLC 200 System (Eksigent; Redwood City, CA, USA). Two microliters of each sample was injected on a Luna 5 μm, 150 mm × 4.6 mm HPLC column (Phenomenex, Torrance, CA). The mobile phases were: (A) 0.01% heptafluorobutyric acid, 0.1% formic acid in water, and (B) 0.01%heptafluorobutyric acid, 0.1% formic acid in methanol. The needle-rinse solvent was methanol. The HPLC flow rate was set at 0.8 mL/min and eluted with a gradient of 2–40% solvent B for 6 min, 40% solvent B for 4 min, 40–90% solvent B for 0.5 min, 90% solvent B for 0.5 min, 90–2% solvent B for 1 min, and followed by 2% solvent B for 3 min. The column was reequilibrated with 2% B for 1 min prior to the next injection. In QTRAP5500, the parameters were set to positive ionization mode, with curtain gas set at 40 psi, nebulizer gas at 60 psi, IonSprayTM voltage at 5500 V, ion source temperature at 600 °C, and CAD gas at medium and to negative ionization mode, with curtain gas set at 40 psi, nebulizer gas at 25 psi, IonSprayTM voltage at −4500 V, ion source temperature at 600 °C, and CAD gas at medium. The MRM transitions for the analyses, declustering potential (DP), entrance potential (EP), collision energy (CE), and the collision cell exit potential (CXP) are listed in Supplementary Table 1. Data processing was carried out using Analyst software (AB SCIEX, version 1.6.1). The lowest limit of quantitation (LLOQ) was determined as the lowest detected concentration with coefficient of variation less than 10%. The instrument lower limit of detection (LLOD) was based on a signal-to-noise value greater than 3. The raw data of MRM was processed using Multiquant software 2.0.2. SignalFinder1 (AB SCIEX) was applied to calculate the corresponding peak areas of MRM signals.

### Statistical Analysis Workflow

The statistical analysis framework included EH biomarker identification, clinical effect evaluation and biomarker validation according to clinical outcomes.

- Metabolic biomarker identification-For metabolomics data, the multivariate analysis and univariate analysis were performed as an integral to determine the most important plasma metabolites for EH and its treatment, or namely, the biomarkers. Pareto-scaled data were Partial least square discriminant analyzed (PLS-DA) by SIMCA-P (version 11.0; Umetrics, Umeå, Sweden) and MetaboAnalyst (http://www.metaboanalyst.ca/MetaboAnalyst/) to find differential metabolites between groups. Score scatter plot and loading plot visualized the separation of samples and the metabolites which were responsible for the separation. Data were also preprocessed using orthogonal signal correction (OSC) which was generally used in metabonomic analysis to filter the unrelated variations. Variable Importance Plot (VIP) that indicated the metabolites changed dramatically in different groups were identified and those whose VIP > = 1.0 were considered to be the key biomarker in this stage. The paired t test (P < 0.05) and fold-change (FC value > 1.2 or <0.8) (by OriginPro, version 9.0, OriginLab Corporation, Northampton, MA 01060 USA) were also performed to strength the above results.
- Clinical effect evaluation- clinical data and the effect of acupuncture were analyzed by paired t test (Graph Prism 5.0 software package (Version 5.01; GraphPad software, San Diego California USA, www.graphpad.com).
- Clinical importance validation of the biomarkers- in this stage, a bivariate correlation (Pearson correlation) was used to analyze the correlation between the level of key metabolites and the clinical outcomes of EH.

## Additional Information

**How to cite this article**: Yang, M. *et al.* A Targeted Metabolomics MRM-MS Study on Identifying Potential Hypertension Biomarkers in Human Plasma and Evaluating Acupuncture Effects. *Sci. Rep.* **6**, 25871; doi: 10.1038/srep25871 (2016).

## Supplementary Material

Supplementary Information

## Figures and Tables

**Table 1:** Demographic information on Hypertension Patients vs. Healthy Controls.

Baseline	Hypertension (n = 113)	Control (n = 15)	*P* value
Demographics			
Female (%)	69.23%	80%	/
Age (years)	59.82 ± 8. 95	56.33 ± 7.73	0.2987
Physical Examination			
24-hour SBP^∇^ (mm Hg)	145.1 ± 9.28	113.6 ± 9.00	<0.0001**
24-hour DBP^Δ^ (mm Hg)	88.35 ± 7.92	75.14 ± 5.98	<0.0001**
BMI (kg/m^2^)	25.77 ± 3.74	24. 29 ± 0.81	0.1693
Blood sugar (mmol/L)	5.04 ± 0.30	4.99 ± 0.42	0.752
Total Cholesterol (mmol/L)	4.82 ± 0.55	4.52 ± 0.41	0.149
RBC count (×10^12^/L)	4.48 ± 0.48	4.49 ± 0.57	0.958
WBC count (×10^9^/L)	5.13 ± 0.93	5.18 ± 1.41	0.917
ALT (unit/L)	33.40 ± 11.01	30.33 ± 5.39	0.362
AST (unit/L)	28.40 ± 7.72	26.07 ± 9.36	0.520
BUN (mmol/L)	5.64 ± 1.60	5.33 ± 1.52	0.650
sCr (μmol/L)	72.36 ± 10.82	73.00 ± 11.77	0.897
Serum uric acid (μmol/L)	258.54 ± 81.78	241.03 ± 37.22	0.486
HDL-C (mmol/L)	1.13 ± 0.25	1.19 ± 0.27	0.557
LDL-C (mmol/L)	2.89 ± 0.43	2.60 ± 0.41	0.112

∇: Systolic blood pressure; Δ: Diastolic blood pressure; ^**^P < 0.01 represents significant difference.

**Table 2:** Biological properties of OA and MI, and correlation of target metabolite with blood pressure.

Metabolite	Molecular weight	Pearson Correlations Coefficient				Related biological pathways
		24h SBP	24h DBP	SBP ND	DBP ND	
Myo-inositol	180.16	−0.47	−0.40	0.35	0.12	Galactose metabolism; Ascorbate and aldarate metabolism; Streptomycin biosynthesis; Inositol phosphate metabolism; Metabolic pathways; Biosynthesis of secondary metabolites; Microbial metabolism in diverse environments; ABC transporters; Phosphatidyl inositol signaling system
Oleic acid	282.46	0.50	0.05	−0.44	−0.37	Fatty acid biosynthesis; Cutin, suberine and wax biosynthesis; Biosynthesis of unsaturated fatty acids; Biosynthesis of plant secondary metabolites

**Table 3:** Blood pressure change in hypertension after acupuncture treatment.

Parameters	Baseline (n = 113)	Post-treatment (n = 108)	*P* value
24-hour SBP^∇^	145.1 ± 9.28	140.7 ± 9.59	< 0.0001**
24-hour DBP^Δ^	88.35 ± 7.92	85.86 ± 7.95	0.0024**
Day SBP	147.8 ± 9.77	143.3 ± 10.28	< 0.0001**
Day DBP	90.08 ± 8.25	87.67 ± 8.19	0.0033**
Night SBP	135.6 ± 13.23	132.4 ± 13.04	0.0130*
Night DBP	82.11 ± 8.91	79.93 ± 9.57	0.0474*
SBP Variability (%)	15.22 ± 4.938	16.55 ± 5.352	0.2755
DBP Variability (%)	19.67 ± 8.399	19.51 ± 6.336	0.9326
MAP^▲^	103.5 ± 12.77	102.2 ± 6.302	0.7383
Pulse (beats/min)	71.57 ± 7.068	76.36 ± 8.599	0.0151*
Nocturnal Dipping (SBP)	8.07 ± 8.53	7.52 ± 7.67	0.4267
Nocturnal Dipping (DBP)	8.68 ± 7.90	8.78 ± 7.30	0.9688
SBP mensor ± SE	145.61 ± 0.95	138.50 ± 1.03	< 0.0001
SBP amplitude (95% CL)	13.01 (8.12, 17.91)	7.94 (2.61, 13.26)	/
SBP acrophase (95% CL)	−214.74 (−236.85, −192.63)	−232.89 (−275.08, −270.02)	/
DBP mensor ± SE	84.86 ± 0.65	82.10 ± 0.62	0.0051
DBP amplitude (95% CL)	7.20 (3.85, 10.56)	5.72 (2.51, 8.94)	/
DBP acrophase (95% CL)	−198.30 (−226.09, −180.09)	−243.09 (−277.28, −270.10)	/
Blood sugar (mmol/L)	5.04 ± 0.30	5.02 ± 0.27	0.944
Total Cholesterol (mmol/L)	4.82 ± 0.55	4.73 ± 0.47	0.409
RBC count (×10^12^/L)	4.34 ± 0.18	4.33 ± 0.30	0.952
WBC count (×10^9^/L)	5.16 ± 0.98	4.49 ± 0.84	0.005**
ALT (unit/L)	32.44 ± 11.23	24.67 ± 6.00	0.046*
AST (unit/L)	27.22 ± 7.17	26.56 ± 7.33	0.764
BUN (mmol/L)	5.91 ± 1.43	5.24 ± 1.17	0.029*
sCr (μmol/L)	71.07 ± 10.63	68.59 ± 12.49	0.551
Serum uric acid (μmol/L)	251.9 ± 83.82	254.3 ± 61.56	0.823
HDL-C (mmol/L)	1.17 ± 0.22	1.02 ± 0.15	0.096
LDL-C (mmol/L)	2.87 ± 0.45	2.69 ± 0.40	0.173

∇: Systolic blood pressure; Δ: Diastolic blood pressure; ▲: Mean Arterial Pressure; *P < 0.05; **P < 0.01. Nocturnal Dipping = (daytime BP-nighttime BP)/daytime BP; Reverse-dipper: <0% nocturnal SBP fall; Nondippers: with ≥0% but <10%; Dippers: with ≥10% but <20%; extreme-dippers: with ≥20%.

RBC: red blood cell; WBC: white blood cell; ALT: Alanine Aminotransferase; AST: Aspartate Aminotransferase; BUN: blood urea nitrogen; Scr: HDL-C: high-density lipoprotein cholesterol; LDL-C: low-density lipoprotein cholesterol.

## References

[b1] Messerli F. H., Williams B. & Ritz E. Essential hypertension. Lancet 370, 591–603 (2007). 17707755 10.1016/S0140-6736(07)61299-9

[b2] Ong K. L., Cheung B. M., Man Y. B., Lau C. P. & Lam K. S. Prevalence, awareness, treatment, and control of hypertension among United States adults 1999–2004. Hypertension 49, 69–75 (2007). 17159087 10.1161/01.HYP.0000252676.46043.18

[b3] Gu D. *et al.* Prevalence, awareness, treatment, and control of hypertension in China. Hypertension 40, 920–927 (2002). 12468580 10.1161/01.hyp.0000040263.94619.d5

[b4] Brindle J. T., Nicholson J. K., Schofield P. M., Grainger D. J. & Holmes E. Application of chemometrics to 1 H NMR spectroscopic data to investigate a relationship between human serum metabolic profiles and hypertension. Analyst 128, 32–36 (2003). 12572799 10.1039/b209155k

[b5] García-Puig J. *et al.* Glucose metabolism in patients with essential hypertension. Am. J. Med 119, 318–326 (2006). 16564774 10.1016/j.amjmed.2005.09.010

[b6] Schillaci G. *et al.* Prognostic value of the metabolic syndrome in essential hypertension. J. Am. Coll. Cardiol. 43, 1817–1822 (2004). 15145106 10.1016/j.jacc.2003.12.049

[b7] Flachskampf F. A. *et al.* Randomized trial of acupuncture to lower blood pressure. Circulation 115, 3121–3129 (2007). 17548730 10.1161/CIRCULATIONAHA.106.661140

[b8] Wurtz P. *et al.* Metabolite profiling and cardiovascular event risk: a prospective study of 3 population-based cohorts. Circulation 131, 774–785, 10.1161/circulationaha.114.013116 (2015). 25573147 PMC4351161

[b9] Miura K. *et al.* Relationship of dietary monounsaturated fatty acids to blood pressure: the international study of macro/micronutrients and blood pressure. J. Hypertens. 31, 1144 (2013). 23572200 10.1097/HJH.0b013e3283604016 PMC4109685

[b10] Gremmels H. *et al.* Oleic acid increases mitochondrial reactive oxygen species production and decreases endothelial nitric oxide synthase activity in cultured endothelial cells. Eur. J. Pharmacol. 751, 67–72, 10.1016/j.ejphar.2015.01.005 (2015). 25595727

[b11] Yang Q. *et al.* Influence of the membrane lipid structure on signal processing via G protein-coupled receptors. Mol. Pharmacol. 68, 210–217 (2005). 15837842 10.1124/mol.105.011692

[b12] Funari S. S., Barceló F. & Escribá P. V. Effects of oleic acid and its congeners, elaidic and stearic acids, on the structural properties of phosphatidylethanolamine membranes. J. Lipid Res. 44, 567–575 (2003). 12562874 10.1194/jlr.M200356-JLR200

[b13] Croze M. L. & Soulage C. O. Potential role and therapeutic interests of myo-inositol in metabolic diseases. Biochimie 95, 1811–1827 (2013). 23764390 10.1016/j.biochi.2013.05.011

[b14] D’Anna R. *et al.* Effects of a New Flavonoid and Myo-Inositol Supplement on Some Biomarkers of Cardiovascular Risk in Postmenopausal Women: A Randomized Trial. Int. J. Endocrinol. 2014, 7, 10.1155/2014/653561 (2014). PMC4164131 25254044

[b15] Abou-Saleh H. *et al.* Inositol 1,4,5-Trisphosphate (IP(3)) Receptor Up-regulation in Hypertension Is Associated with Sensitization of Ca(2+) Release and Vascular Smooth Muscle Contractility. J. Biol. Chem. 288, 32941–32951, 10.1074/jbc.M113.496802 (2013). 24097979 PMC3829145

[b16] Greene E. L., Lu G., Zhang D. & Egan B. M. Signaling Events Mediating the Additive Effects of Oleic Acid and Angiotensin II on Vascular Smooth Muscle Cell Migration. Hypertension 37, 308–312, 10.1161/01.hyp.37.2.308 (2001). 11230290

[b17] Malvasi A. *et al.* Myo-inositol, D-chiro-inositol, folic acid and manganese in second trimester of pregnancy: a preliminary investigation. Eur. Rev. Med. Pharmacol. Sci. 18, 270–274 (2014). 24488919

[b18] D’Anna, Sr, R. *et al.* [11-OR]: Myo-inositol in the prevention of gestational diabetes and its complications. Pregnancy Hypertens. 5, 6 (2015).

[b19] D’Anna R. *et al.* myo-Inositol Supplementation and Onset of Gestational Diabetes Mellitus in Pregnant Women With a Family History of Type 2 Diabetes A prospective, randomized, placebo-controlled study. Diabetes care 36, 854–857 (2013). 23340885 10.2337/dc12-1371 PMC3609506

[b20] Trialists’ Collaboration B. P. L. T. *et al.* Effects of different regimens to lower blood pressure on major cardiovascular events in older and younger adults: meta-analysis of randomised trials. BMJ 336, 1121–1123 (2008). 18480116 10.1136/bmj.39548.738368.BE PMC2386598

[b21] Feldstein C. A. Lowering blood pressure to prevent stroke recurrence: a systematic review of long-term randomized trials. J. Am. Soc. Hypertens. 8, 503–513, 10.1016/j.jash.2014.05.002 (2014). 25064772

[b22] Thomopoulos C., Parati G. & Zanchetti A. Effects of blood pressure lowering on outcome incidence in hypertension: 4. Effects of various classes of antihypertensive drugs–overview and meta-analyses. J Hypertens 33, 195–211, 10.1097/hjh.0000000000000447 (2015). 25485720

[b23] Meschia J. F. *et al.* Guidelines for the Primary Prevention of Stroke: A Statement for Healthcare Professionals From the American Heart Association/American Stroke Association. Stroke 45, 3754–3832, 10.1161/str.0000000000000046 (2014). 25355838 PMC5020564

[b24] Yin J. *et al.* Hypoglycemic effects and mechanisms of electroacupuncture on insulin resistance. Am. J. Physiol. Regul. Integr. Comp. Physiol. 307, R332–R339 (2014). 24848362 10.1152/ajpregu.00465.2013 PMC4121630

[b25] Lin R. T. *et al.* Electroacupuncture improves glucose tolerance through cholinergic nerve and nitric oxide synthase effects in rats. Neurosci. Lett. 494, 114–118, 10.1016/j.neulet.2011.02.071 (2011). 21376780

[b26] Zhu L. L., Wei W. M., Zeng Z. H. & Zhuo L. S. [Impact of electro-acupuncture on lipid metalolism in rats with non-alcoholic fatty liver disease]. Sichuan Da Xue Xue Bao Yi Xue Ban. 43, 847–850 (2012). 23387211

[b27] Xu T. & Li Z. R. [Progress of studies on effects of acupuncture on cellular signal transduction]. Zhen Ci Yan Jiu. 36, 150–154 (2011). 21717785

[b28] Li J. *et al.* Electroacupuncture improves cerebral blood flow and attenuates moderate ischemic injury via Angiotensin II its receptors-mediated mechanism in rats. BMC Complement. Altern. Med. 14, 441, 10.1186/1472-6882-14-441 (2014). 25387826 PMC4237754

[b29] Sun D. W., Du Y. H. & Shi L. [Effect of electroacupuncture on inositol triphosphate and diacylglycerol levels in cerebral arteries of cerebral ischemia rats]. Zhen Ci Yan Jiu. 33, 392–396 (2008). 19288900

[b30] Association W. M. World Medical Association Declaration of Helsinki. Ethical principles for medical research involving human subjects. Bull. World Health Organ. 79, 373 (2001). 11357217 PMC2566407

[b31] Davidson M. A review of the current status of the management of mixed dyslipidemia associated with diabetes mellitus and metabolic syndrome. Am. J. Cardiol. 102, 19L–27L (2008). 19084086 10.1016/j.amjcard.2008.09.071

[b32] Umemoto T. *et al.* Apolipoprotein AI and high-density lipoprotein have anti-inflammatory effects on adipocytes via cholesterol transporters ATP-binding cassette A-1, ATP-binding cassette G-1, and scavenger receptor B-1. Circ. Res. 112, 1345–1354 (2013). 23501697 10.1161/CIRCRESAHA.111.300581 PMC3767575

[b33] Douard V. & Ferraris R. P. The role of fructose transporters in diseases linked to excessive fructose intake. J. Physiol. 591, 401–414 (2013). 23129794 10.1113/jphysiol.2011.215731 PMC3577529

[b34] Calderón-Santiago M., Priego-Capote F., Galache-Osuna J. & de Castro M. L. Method based on GC–MS to study the influence of tricarboxylic acid cycle metabolites on cardiovascular risk factors. J. Pharm. Biomed. Anal. 74, 178–185 (2013). 23245249 10.1016/j.jpba.2012.10.029

[b35] Bispo J. A. M., de Sousa Vieira E. E., Silveira L. & Fernandes A. B. Correlating the amount of urea, creatinine, and glucose in urine from patients with diabetes mellitus and hypertension with the risk of developing renal lesions by means of Raman spectroscopy and principal component analysis. J. Biomed. Opt 18, 087004–087004 (2013). 10.1117/1.JBO.18.8.087004 23929457

[b36] Pinto V., Pinho M. J. & Soares-da-Silva P. Renal amino acid transport systems and essential hypertension. FASEB J. 27, 2927–2938 (2013). 23616567 10.1096/fj.12-224998

[b37] Terés S. *et al.* Oleic acid content is responsible for the reduction in blood pressure induced by olive oil. Proc. Natl. Acad. Sci. USA 105, 13811–13816 (2008). 18772370 10.1073/pnas.0807500105 PMC2544536

[b38] Hirose M. *et al.* Imbalance of renal production between 5-hydroxytryptamine and dopamine in patients with essential hypertension complicated by microalbuminuria. Am. J. Hypertens. 26, 227–233 (2013). 23382407 10.1093/ajh/hps008

