# Supplementary material for: A Targeted Metabolomics MRM-MS Study on Identifying Potential Hypertension Biomarkers in Human Plasma and Evaluating Acupuncture Effects
Source: Sci Rep. 2016 May 16;6:25871. doi: 10.1038/srep25871 (PMC4867614; doi:10.1038/srep25871)

**A Targeted Metabolomics MRM-MS Study on Identifying Potential Hypertension Biomarkers in Human Plasma and Evaluating Acupuncture Effects**

**Mingxiao Yang^1^*, Zheng Yu^1^*, Shufang Deng^1^, Xiaomin Chen^2^, Liang Chen^1^, Zhenyu Guo^2^, Hui Zheng^1^, Lin Chen^1^, Dingjun Cai^1^, Bo Wen^2^, Qiaofeng Wu^1†^, and Fanrong Liang^1†^**

## Supplemental Tables

## Table1. The MRM transitions for the analytes , declustering potential (DP), entrance potential (EP), collision energy (CE) and the collision cell exit potential(CXP)

| **Q1** | **Q3** | **Analyte** | **DP** | **EP** | **CE** | **CXP** |
| --- | --- | --- | --- | --- | --- | --- |
| 118.5 | 58.9 | Betaine_1 | 200 | 10 | 28.4 | 13 |
| 118.5 | 73.1 | Betaine_2 | 100 | 10 | 25 | 13 |
| 117.7 | 42 | Hexanoic acid_1 | 200 | 10 | 70.67 | 13 |
| 133.1 | 70 | Oxaloacetic acid_1 | 100 | 10 | 24.2 | 13 |
| 133.1 | 87 | Oxaloacetic acid_2 | 200 | 10 | 14.9 | 13 |
| 183.1 | 69 | D-Sorbitol_1 | 100 | 10 | 19.94 | 13 |
| 183.1 | 129 | D-Sorbitol_2 | 100 | 10 | 11.79 | 13 |
| 183.1 | 147 | D-Sorbitol_3 | 100 | 10 | 10.63 | 13 |
| 161.3 | 69.1 | Pimelic acid_1 | 100 | 10 | 22.02 | 13 |
| 137 | 55 | Hypoxanthine_1 | 250 | 10 | 43 | 10 |
| 137 | 81.9 | Hypoxanthine_2 | 200 | 10 | 33 | 10 |
| 137 | 119 | Hypoxanthine_3 | 230 | 10 | 28 | 10 |
| 120.7 | 57 | D-Homoserine_1 | 60 | 10 | 23.49 | 13 |
| 120.7 | 75.1 | D-Homoserine_2 | 70 | 10 | 14.95 | 13 |
| 169 | 141 | Uric acid _1 | 130 | 10 | 21.75 | 13 |
| 169 | 152.1 | Uric acid _2 | 130 | 10 | 23.45 | 13 |
| 169 | 70.1 | Uric acid _3 | 130 | 10 | 31.34 | 13 |
| 132 | 86 | Isoleucine _1 | 50 | 10 | 14.9 | 8 |
| 132 | 69.1 | isoleucine _2 | 50 | 10 | 23.3 | 8 |
| 132 | 44.1 | isoleucine _3 | 50 | 10 | 32.1 | 8 |
| 182.1 | 119 | L-Tyrosine_1 | 80 | 10 | 23.76 | 13 |
| 182.1 | 136.1 | L-Tyrosine_2 | 40 | 10 | 18.1 | 8 |
| 182.1 | 165.1 | L-Tyrosine_3 | 40 | 10 | 13.1 | 8 |
| 166.1 | 103.1 | L-Phenylalanine_1 | 100 | 10 | 35 | 13 |
| 166.1 | 77.1 | L-Phenylalanine_2 | 128 | 10 | 50.2 | 8 |
| 166.1 | 120 | L-Phenylalanine_3 | 50 | 10 | 31.7 | 8 |
| 205 | 146 | L-Tryptophan_1 | 150 | 10 | 23.65 | 13 |
| 205 | 118 | L-Tryptophan_2 | 45 | 10 | 34.4 | 8 |
| 205 | 115.1 | L-Tryptophan_3 | 45 | 10 | 49 | 8 |
| 132.1 | 86.1 | L-Leucine_1 | 50 | 10 | 14.9 | 8 |
| 132.1 | 43.1 | L-Leucine_2 | 100 | 10 | 35.1 | 13 |
| 132.1 | 44.1 | L-Leucine_3 | 50 | 10 | 29.5 | 8 |
| 120.1 | 102.1 | L-Threonine_1 | 80 | 10 | 15.07 | 8 |
| 120.1 | 56 | L-Threonine_2 | 30 | 10 | 24 | 8 |
| 120.1 | 74 | L-Threonine_3 | 30 | 10 | 14 | 8 |
| 118.1 | 57 | L-Vline_1 | 100 | 10 | 12.01 | 13 |
| 118.1 | 72.1 | L-Vline_2 | 40 | 10 | 15.8 | 8 |
| 118.1 | 55.1 | L-Vline_3 | 40 | 10 | 29.6 | 8 |
| 76 | 30 | Glycine_1 | 50 | 10 | 24.8 | 8 |
| 76 | 48 | Glycine_2 | 50 | 10 | 11.2 | 8 |
| 61.4 | 44 | urea_1 | 100 | 10 | 24.17 | 13 |
| 61.4 | 45 | urea_2 | 100 | 10 | 26.21 | 13 |
| 175.5 | 114.1 | Citrulline_1 | 70.92 | 10 | 21 | 13 |
| 176.1 | 159 | Citrulline_2 | 40 | 10 | 15 | 8 |
| 176.1 | 70.1 | Citrulline_3 | 40 | 10 | 28.8 | 8 |
| 341.2 | 178.9 | Sucrose_1 | -100 | -10 | -16.8 | -21 |
| 341.2 | 89 | Sucrose_2 | -100 | -10 | -28 | -21 |
| 341.2 | 119 | Sucrose_3 | -100 | -10 | -21 | -21 |
| 178.8 | 58.9 | D-(+)-Galactose_1 | -100 | -10 | -22 | -22 |
| 178.8 | 70.7 | D-(+)-Galactose_2 | -100 | -10 | -23 | -23 |
| 178.8 | 88.6 | D-(+)-Galactose_3 | -100 | -10 | -11 | -11 |
| 178.8 | 160.9 | myo-Inositol_1 | -100 | -10 | -15.01 | -13 |
| 178.8 | 87 | myo-Inositol_2 | -100 | -10 | -22.95 | -13 |
| 178.8 | 116.8 | myo-Inositol_3 | -100 | -10 | -19.01 | -13 |
| 179.1 | 58.8 | D-(−)-Fructose_1 | -80 | -10 | -17 | -13 |
| 179.1 | 71 | D-(−)-Fructose_2 | -80 | -10 | -16 | -13 |
| 179.1 | 89 | D-(−)-Fructose_3 | -80 | -10 | -11 | -13 |
| 341.3 | 161 | D-(+)-Cellobiose_1 | -100 | -10 | -9.59 | -21 |
| 341.3 | 179 | D-(+)-Cellobiose_2 | -100 | -10 | -10.41 | -21 |
| 341.3 | 101 | D-(+)-Cellobiose_3 | -100 | -10 | -19.88 | -21 |
| 145.3 | 57.1 | α-ketoglutaric acid_1 | -10 | -14.95 | -14 | -14 |
| 145.3 | 73 | α-ketoglutaric acid_2 | -10 | -19.34 | -14 | -14 |
| 145.3 | 101.2 | α-ketoglutaric acid_3 | -10 | -18 | -14 | -14 |

## Supplemental Figures

Figure 1: The variable importance in the projection (VIP) score of OA and MI and their concentration in three groups. (a) VIP scores of OA and MI; (b) concentrations of critical metabolites in baseline, post-treatment and healthy control.


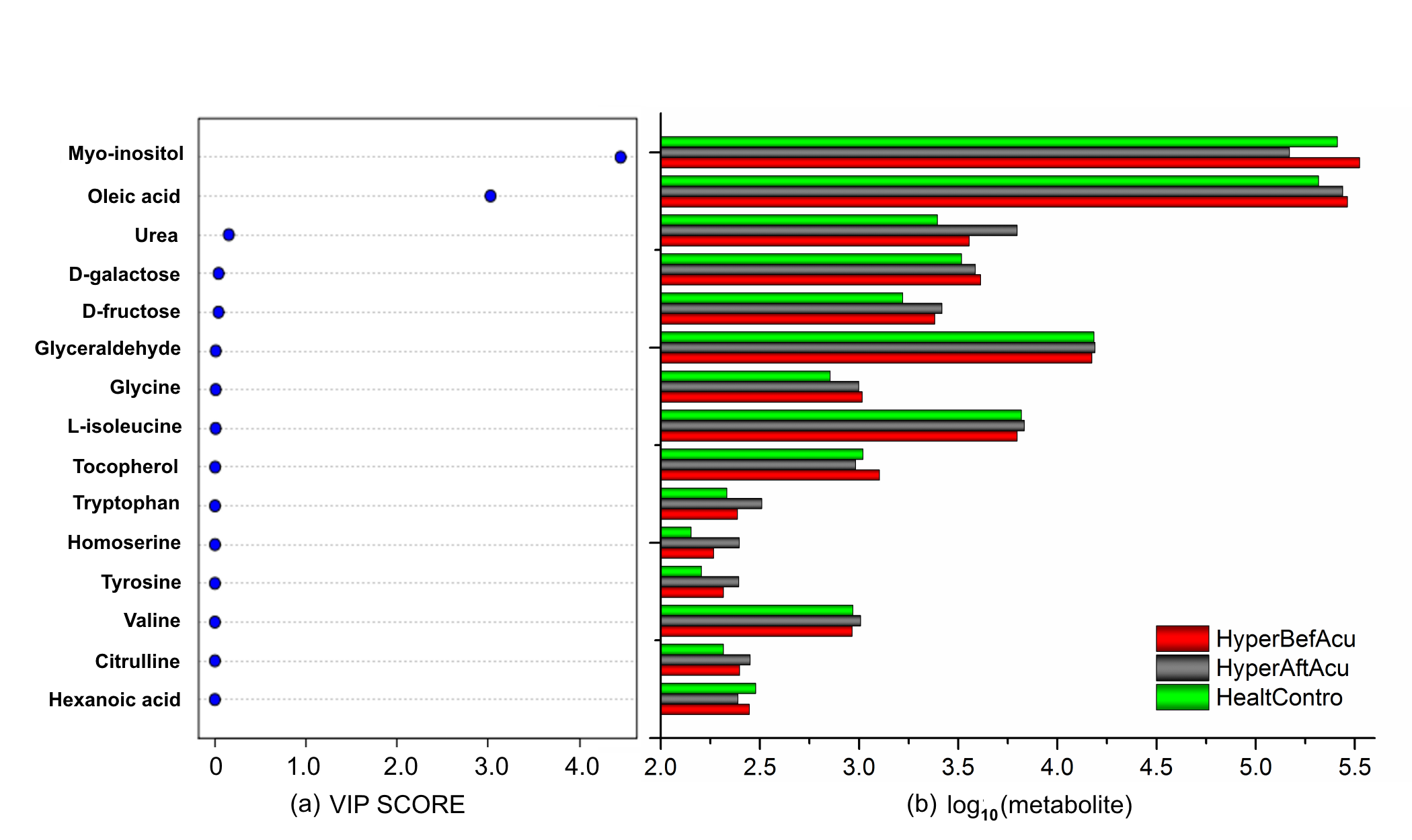


Figure 2: Normalized metabolite concentration for hypertension patients and healthy subjects. This figure illustrated the median-normalized, Log10-transormed metabolite concentration for several targeted metabolites. A is abbreviated for after treatment, B is short for before treatment, and C for control.


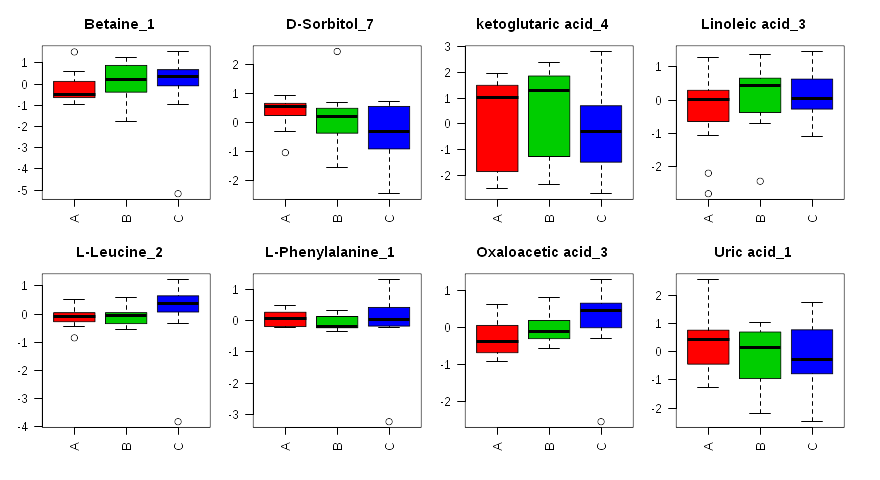


Figure 3: OA related metabolic pathway


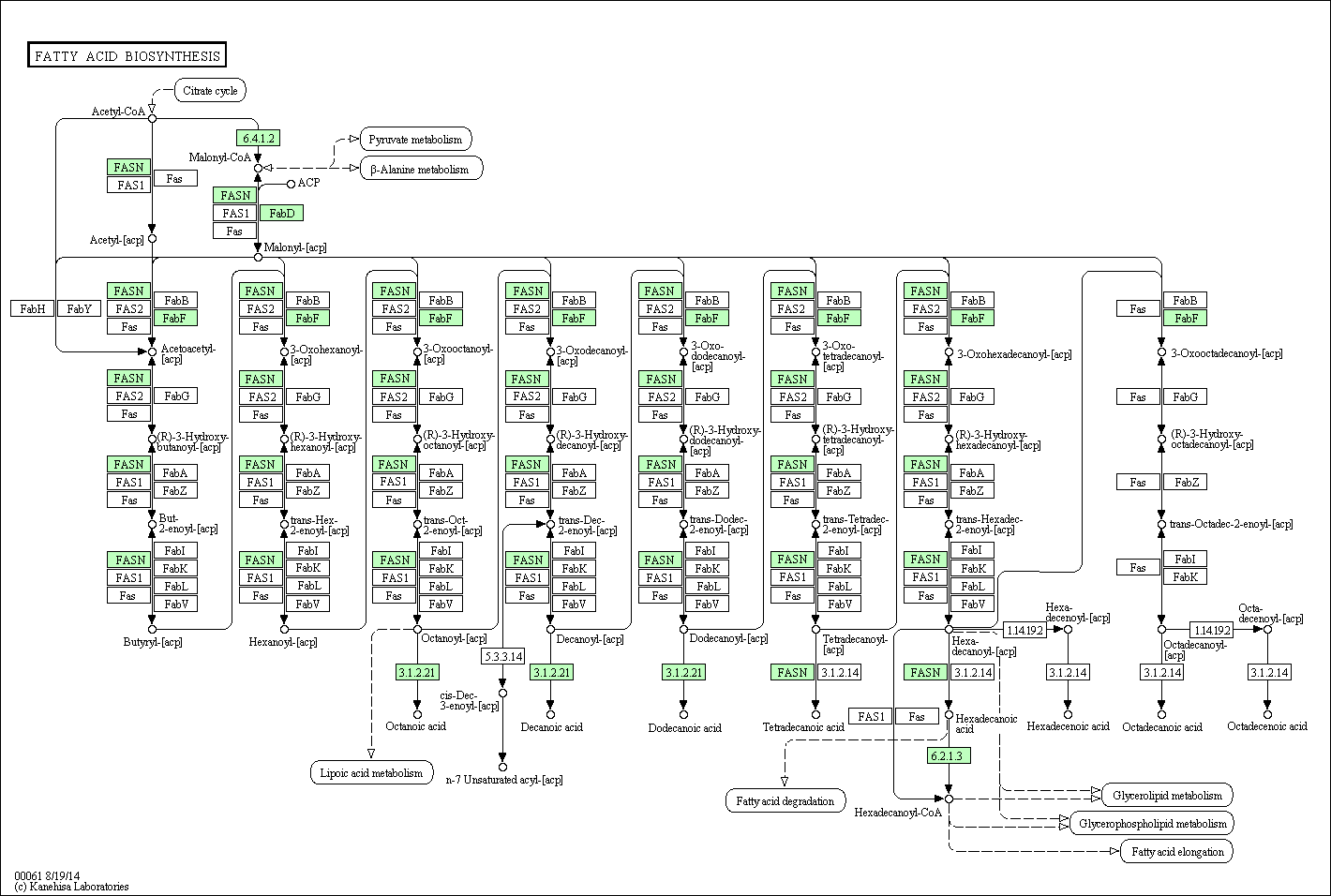


Figure 4: MI related metabolic pathway


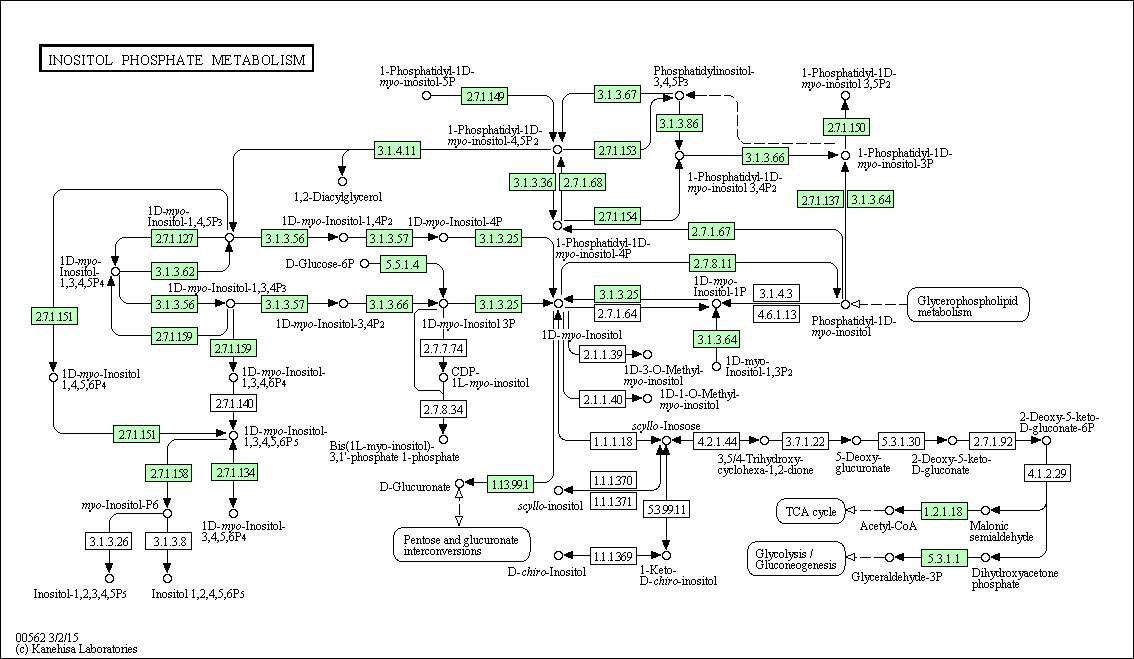

Supplement: Supplementary Information [file srep25871-s1.doc]
